# Conservation of resources theory and research use in health systems

**DOI:** 10.1186/1748-5908-5-79

**Published:** 2010-10-20

**Authors:** Celeste Alvaro, Renée F Lyons, Grace Warner, Stevan E Hobfoll, Patricia J Martens, Ronald Labonté, Richard E Brown

**Affiliations:** 1 Atlantic Health Promotion Research Centre, Faculty of Health Professions, Dalhousie University, Canada; 2 Bridgepoint Collaboratory for Research and Innovation, Bridgepoint Health, University of Toronto; Atlantic Health Promotion Research Centre, Dalhousie University, Canada; 3 School of Occupational Therapy, Dalhousie University, Canada; 4 Department of Behavioral Sciences at Rush University and Medical College, USA; 5 Manitoba Centre for Health Policy, Department of Community Health Sciences, Faculty of Medicine, University of Manitoba, Canada; 6 Institute of Population Health, Department of Epidemiology and Community Medicine, University of Ottawa, Canada; 7 UCLA Center for Health Policy Research, School of Public Health, University of California, USA

## Abstract

**Background:**

Health systems face challenges in using research evidence to improve policy and practice. These challenges are particularly evident in small and poorly resourced health systems, which are often in locations (in Canada and globally) with poorer health status. Although organizational resources have been acknowledged as important in understanding research use resource theories have not been a focus of knowledge translation (KT) research. What resources, broadly defined, are required for KT and how does their presence or absence influence research use?

In this paper, we consider conservation of resources (COR) theory as a theoretical basis for understanding the capacity to use research evidence in health systems. Three components of COR theory are examined in the context of KT. First, resources are required for research uptake. Second, threat of resource loss fosters resistance to research use. Third, resources can be optimized, even in resource-challenged environments, to build capacity for KT.

**Methods:**

A scan of the KT literature examined organizational resources needed for research use. A multiple case study approach examined the three components of COR theory outlined above. The multiple case study consisted of a document review and key informant interviews with research team members, including government decision-makers and health practitioners through a retrospective analysis of four previously conducted applied health research studies in a resource-challenged region.

**Results:**

The literature scan identified organizational resources that influence research use. The multiple case study supported these findings, contributed to the development of a taxonomy of organizational resources, and revealed how fears concerning resource loss can affect research use. Some resources were found to compensate for other resource deficits. Resource needs differed at various stages in the research use process.

**Conclusions:**

COR theory contributes to understanding the role of resources in research use, resistance to research use, and potential strategies to enhance research use. Resources (and a lack of them) may account for the observed disparities in research uptake across health systems. This paper offers a theoretical foundation to guide further examination of the COR-KT ideas and necessary supports for research use in resource-challenged environments.

## Background

Knowledge translation (KT) is the 'exchange, synthesis, and ethically-sound application of knowledge -- within a complex system of interactions among researchers and users -- to accelerate the capture of the benefits of research through improved health, more effective services and products, and a strengthened healthcare system' [1]. Accordingly, KT spans all steps in between the creation of knowledge and its application to benefit society, with an emphasis on effective partnerships among researchers and users. In practice, KT strategies may involve activities to ensure that research evidence is available and used in decision-making to determine policies, programs, and practices to improve health. Like any change process, KT requires resources and the elasticity that is afforded by their availability. Given this proposition, what insight does the KT literature offer concerning research use in resource-challenged environments? Over the past 10 years, considerable effort has been placed on KT and evidence-based decision-making and in understanding and improving capacity for research use within health systems (*i.e*., federal health departments, provincial or state departments of health, district or regional health authorities, hospitals, community health organizations [2,3]). Despite the growing number of frameworks [4-9], strategies [10-14], and investments in KT [15-17], there has been limited research in the development of explanations of research use. Research examining theory-based approaches to KT (*e.g*., cognitive and behavioural change) has been mainly applied to changing clinical practitioner behaviour [18-20] rather than health systems. However, the work of Dobson and Fitzgerald [21], Lavis *et al*. [22-25], and Kitson [26] has contributed to an increased understanding about the challenges of using evidence, approaches to using research evidence, and the organizational support that is necessary for research use in resource-challenged environments.

Research that examines KT in the context of developing countries suggests that there is substantial variation in the capacity for research uptake among health systems [27-29]. Low- and middle-income countries often lack the human and financial resource capacity to act on research evidence as they struggle to keep up with basic healthcare demands. At minimum, research uptake requires functioning health systems and an adequate number of skilled health workers [30]. Within developed countries, resources are also thought to be stretched beyond capacity as systems are pressed to do more with less, and healthcare costs continue to rise with our aging population and increasing rates of chronic disease [31,32]. Resources are not limited to money, objects, and people. Values, skills, conditions, and culture are resources by virtue of their value in effecting change and facilitating the acquisition of additional resources. Although resources such as values, skills, conditions, and culture are seemingly more intangible in comparison to more tangible resources with a physical presence, they serve to build resiliency and provide a degree of elasticity necessary to adapt to change. Resource scarcity can enhance resistance to using evidence to change policy and practice and drive people to conserve existing resource pools [33]. A lack of resources, or the threat of losing existing resources, may limit receptivity and responsiveness to research within health systems. Consequently, operating below specific resource thresholds may contribute to a widening 'health systems gradient' wherein organizations with fewer resources fall further behind organizations with greater resources.

A perceived lack of resources may have important consequences for research use in addition to the actual lack of resources. Health systems managers and staff face concurrent demands of using evidence to improve the quality of patient care, within the parameters of accountability and cost-effectiveness. New research evidence can be perceived as a threat to the *status quo *because it must be incorporated within existing structures, often without increased resources to institute change. Research in the nursing context, in particular, has identified perceived barriers to research use (including resource deficits) that lead to the resentment of policy and/or practice changes implicated in the emerging research evidence [34-42]. As evident in the stress and coping literature, individuals and groups become increasingly aversive to risk and show bias in favor of conservation in the face of stress [43]. Given that stress, and resistance to change, is elevated in resource-challenged environments, a greater understanding of the underlying mechanisms by which resources contribute to research use is needed.

### Resource theories

Resource theories offer the potential to understand the role of organizational resources in the uptake of research evidence. Resource theories are based on the premise that a minimum resource threshold is necessary for performance, with increasing difficulty arising as demands increase and outweigh the available resource pools [44]. Resource theories have a long history and span several disciplines, including: cognitive psychology [45-47], biology [48], ecology [49], social psychology [50,51], community psychology [52], economics [53], and sociology [54,55]. Although researchers have adapted resource theories to understand seemingly disparate phenomena, a constant theme across all disciplines is that resources are key determinants of performance, adaptation, and change.

### Conservation of resources theory

In contrast to other resources theories, conservation of resources (COR) theory is of particular interest in understanding research use because it goes beyond merely linking resources to performance. COR theory [56,57] emerged from resource and psychosocial theories of stress and human motivation. Social scientists who study stress have found that personal resources (*e.g*., perceived control, self-efficacy, perceptions of improvement) and social resources (*e.g*., emotional support, assistance from friends and family) buffer against the potential negative impact of stressful life events [58-61]. COR theory extends prior theories by acknowledging that stress stems from the combined effect of the subjective perception of an event as taxing or exceeding available resources [62-64] and the objective or actual environmental circumstances that threaten or cause depletion of people's resources [65-67].

COR theory has been used as an explanatory model for organizational stress in health systems and other organizations [68-75]. COR theory [76,77] may also contribute to understanding the function of resources in KT and how perceived or actual resource constraints affect research use in health systems. The main principles and corollaries of COR theory have been reviewed extensively elsewhere [78]. For the purposes of our research, we extracted three themes of COR theory (Table 1) that are of particular relevance in understanding limitations in capacity to using research and building the resilience for health systems change in resource-challenged environments.

**Table 1:** COR theory themes

**COR theory theme one**:	Resources are required for adaptation and change
**COR theory theme two**:	The threat of loss leads to the protection of assets
**COR theory theme three**:	Resources must be optimized for adaptation

#### Theme one: Resources are required for adaptation and change

In COR theory, resources are defined as objects, conditions, personal characteristics, and energies that are either themselves valued for survival, directly or indirectly, or that serve as a means of achieving these resources [79-82]. Object resources have a physical presence (*e.g*., clothing, shelter). Condition resources are structures or states (*e.g*., status at work, good health) that allow access to or the possession of other resources. Personal resources include skills and traits (*e.g*., occupational skills, self-esteem). Energy resources (*e.g*., money, knowledge) are those whose value is derived from their ability to be exchanged for other resources. It seems reasonable to predict that organizational resources may affect health systems capacity for research use in the same way that resources affect adaptation in individuals, groups, communities, and organizations.

Although the concept of stages of change was not outlined in Hobfoll's COR theory [83], various stage based models of change suggest that some types of resources may be more important than others and that some resources may be more important at different stages of the implementation process than others [84-87].

#### Theme two: The threat of loss leads to the protection of assets

Individuals and groups are threatened by the potential or actual loss of resources, and are therefore motivated to obtain, retain, foster, and protect valued resources for anticipated future needs [88,89]. Those with fewer resources are more vulnerable to resource loss, less capable of resource gain, and highly risk-averse so they often opt to maintain existing resources rather than risk resource depletion [90-93]. Research has shown that, although they are generally in favour of research use, individuals and groups within resource-challenged health systems conserve resources for everyday and future 'rainy day' challenges [94]. Implementing research evidence takes resources and can have considerable implications for policy and practice. Understandably, threat can serve to increase risk aversion, to amplify resistance to change, and to limit action on research evidence.

#### Theme three: Resources must be optimized for adaptation

According to Hobfoll [95], the impact of resource loss far outweighs the impact of equivalent resource gain. Nonetheless, individuals and social units (including systems) with greater resources are often less vulnerable to resource loss, more capable of resource gain, and more 'elastic' (*i.e*., able to take risks) than their resource-challenged counterparts. Therefore, resources must be invested to gain additional resources and to offset the potential or actual loss of resources [96]. Although initially biased in favour of resource conservation, individuals and social units can direct themselves to enhance resources. Strategic resource investment, resource manipulation, resource mobilization (*i.e*., employing resources one possesses or calling upon resources available within one's environment), and resource substitutions (*i.e*., using specific resources in one domain to compensate for a lack of resources in another domain) are important in bolstering capacity for research use [97].

COR theory has recently been applied to the study of how communities cope with natural disaster [98], and terrorism [99], as well as how individuals within organizations cope with occupational stress [100-105]. The evidence in support of COR theory as it relates to resource-challenged regions' capacity to cope with natural disaster (*e.g*., drought) is particularly revealing. Resource-challenged regions continually operate in a state of depleted resources. When an external event (*i.e*., natural disaster) occurs, the event creates added stress on the system and causes a change in the level of resources available [106,107]. Still, some regions that are repeatedly affected by disaster do demonstrate remarkable resilience. Such resilience is, in part, due to pro-active coping interventions aimed at buffering against the negative impact of stress, such as assessing resource-related capacity to cope with stress, fostering preparedness before resources are strained, or increasing resource pools within the community or organization.

In this research, we conducted a scan of the KT literature to identify organizational resources that contribute to research use and examined the three components of COR theory via a multiple-case study. The purpose, methods, and results of the scan and multiple-case study are described in turn, followed by a discussion of the overall findings and potential contributions of our research.

## Methods

### Identifying organizational resources

#### Search Methods

Relevant databases (such as PubMed, Psych Info, Web of Science) were searched using search terms that were agreed upon by lead author and principal investigators. The following key terms (or a combination thereof) were included: knowledge translation, knowledge transfer, knowledge exchange, knowledge utilization/use/uptake, research utilization/use/uptake, barriers to [key term], and facilitators of [key term].

### Inclusion and exclusion criteria

No limitations were placed on publication date (the search was conducted between 2006 and 2008). Publication bibliographies were searched to identify additional literature. Online resources (such as funding agency websites, and websites of academic research centres with a focus on KT), and grey literature on KT in health systems were also included. The initial search yielded approximately 1,200 articles. The articles were reduced to include only those published in English language peer-reviewed journals that were related to organizational and/or systems level research uptake (approximately 100 articles). The articles were themed according to theoretical papers, literature reviews, research studies (including quantitative and qualitative), and commentaries. It should be noted that the majority of articles were descriptive in nature.

#### Search results

There was remarkable consistency in the types of resources identified in the literature. The scan resulted in the generation of an extensive list of organizational resources that contribute to research use (See items in Additional file 1: Table s1 that are identified with the subscript_a_[108-412]).

#### Multiple-case study

A multiple-case study [413,414] that consisted of key informant interviews was designed to confirm the list of resources derived from the literature, identify additional organizational resources, and develop a taxonomy of organizational resources required for research use. This method allowed for an initial exploration of the COR theory themes and their relevance in health systems.

#### Selection of cases

A case was defined as a collaborative research initiative between an academic research centre and a health policy or healthcare organization. Following an initial review of potential cases, four cases were selected. The four selected cases included diverse team members (*i.e*., researchers, practitioners, voluntary agencies, and government), represented varying time frames that ranged from short-term (*i.e*., one year or less) to long-term (*i.e*., multi-year), were initiated because the research evidence indicated that change was necessary (*i.e*., research was identified, synthesized, or conducted), and ranged from having a direct impact on policy and/or practice to having little or no impact on policy and/or practice. The research projects took place in a relatively 'resource-challenged environment' - Atlantic Canada.

### Case one: Urban bikeways

The urban bikeways (UB) project was a relatively short-term (approximately one year) initiative to provide an evidence-based argument for developing safe cycling in an urban region of Canada. A research report that provided a synthesis of research on bikeway systems was carefully developed with decision makers in mind and presented to city council. Researchers actively engaged with municipal staff and city councillors in the research process and the development of a report that was presented to City Council. These activities were instrumental in the establishment of a municipal committee to promote and oversee the development of UBs.

### Case two: Rural stroke services

The rural stroke services (RSS) project was a nationally funded long-term (six year) community alliance for health research to improve stroke prevention and treatment in rural communities, using one community as the unit of analysis. This project consisted of multiple studies including a needs assessment for persons post-stroke, a best practice scan, and asset mapping plus several strategies including community forums, and working groups to develop and implement an evidence-based change strategy.

### Case three: Food cost and security

The food cost and security (FSS) project was a multi-year partnership between an academic research centre, national agencies, and community organizations. The purpose of this research was to build capacity to address the issue of food cost and security at community, provincial and national levels. Project activities included gathering evidence on the cost of food, local advocacy to develop a strategy to impact food security policy, and the use research findings to advocate for broader social change concerning food security.

### Case four: Treatment of depression in rural seniors

The depression in rural seniors (DRS) project was a relatively short-term (one year) project representing a partnership between an academic research centre, affiliated universities, provincial departments of health, a provincial non-profit mental health association, a national mental health association, community organizations, and a subset of local senior citizens. The purpose was to examine access to mental health services for seniors suffering from depression, and to develop a social marketing strategy to encourage seniors to seek mental health services. However, a direct impact on policy or practice was not observed.

### Participants

A letter of invitation requesting their participation in this study was sent to 57 researchers, government representatives, non-governmental organization (NGO) staff, and practitioners affiliated with the four projects described above. A list of individuals who were involved with each of the research projects described above was obtained from the principal investigator. An information sheet describing the research objectives, procedures, and ethics approval was included with the letter of invitation. Two weeks later, participants received a follow-up telephone call to confirm receipt of the information package and their interest in participating in an interview. Face-to-face interviews were then scheduled with 44 participants: 13 health systems policy makers, 11 researchers, 10 clinicians, 9 community health organization representatives, and 1 NGO representative (77% response rate) in the four Atlantic Canadian Provinces. The remaining 23% of those invited declined to participate on the basis of their availability and/or perceived relevance as participants in the study. Those who declined were asked to identify someone who may be more appropriate to contact. There was equal representation of participants across all four cases. The rationale for selecting participants ranging from researchers, policy makers, to practitioners was to ensure that perspectives on research uptake were obtained from individuals across various levels within health systems in partnership with researchers.

### Interview guide and procedures

All procedures and instruments/materials were approved by the university's Human Research Ethics Board. A semi-structured interview guide to examine the COR theory themes was developed and adapted for relevance to each case. The interviews for case studies one, two, and four were conducted by the lead author. The interviews for case study three were conducted by a graduate research assistant who was trained and coached through a series of mock interviews, subtleties of COR theory, and was responsible for coding all interviews. Thus, the level of sophistication in conducting the interviews was comparable across the two interviewers. Interview guides were sent to participants in advance of the interview. Interviews were conducted in person and audiotaped at the participants' workplace. The interviewer began by asking the participant to describe his or her role in the respective project. To assess participants' understanding of resources required for research use and to initiate thinking about resources, participants were asked to identify resources they perceive to be necessary for research use on a general level. Interview questions assessed three central COR-KT themes: Resources are required for adaptation and change; the threat of loss motivates the protection of assets; and resources must be optimized for adaptation.

### COR-KT theme one: Resources are required for adaptation and change (in the context of research)

At the beginning of a semi-structured one-hour interview, participants were asked to indicate the factors (or resources) they believed to be necessary for the uptake of research evidence within health systems (question two). Responses to these questions were compiled and cross-referenced with those found in the literature and were used to develop the taxonomy of organizational resources (see Additional file 1, Table s1).

In keeping with the notion that resource needs may vary as a function of the stages of research uptake (see the overview of COR-KT themes described earlier in this paper), participants were asked to describe the resources available at three points during the research uptake process: the early stages of research uptake, the implementation stage, and the later stages of sustaining newly implemented policies and/or practices (questions three and four).

### COR-KT theme two: The threat of loss leads to the protection of assets

Participants were asked to identify any concerns about resources that arose throughout the course of the project, resource losses associated with research uptake, and actions taken to offset concerns. Participants were also asked to identify actual resource losses and gains that resulted from research uptake, the stage at which losses or gains occurred, and what, if any, actions they engaged in to compensate for the losses or capitalize on the gains (questions six and seven).

### COR-KT theme three: Resources must be optimized for adaptation

Participants were asked to identify what, if any, resources were invested in using research to make changes to policy and/or practice, how these investments differed across the stages of research uptake, and the consequences of these investments (or lack of investment) (question eight). Participants were also asked about how they (or their organization) capitalized on resource strengths and compensated for resource weaknesses (question nine).

### Coding and analysis to develop the taxonomy of organizational resources

Using the composite list in Additional file 1, Table s1, two independent raters grouped similar items, created a category name for each grouping of resources, and identified subcategories within each grouping. Raters then classified each item according to the overall category and the subcategory to which it belonged. Inter-rater reliability, assessed using the intra-class correlation coefficient [415], was r (80) = 0.94, p < 0.01 for the overall category and r (62) = 0.93, p < 0.01 for the component within the overall category. Disagreements between raters typically reflected the somewhat overlapping nature of the categories of resources and were resolved through discussion. The items were then arranged into an initial taxonomy of organizational (health systems) resources that are perceived by the literature and the respondents to influence research use.

### Coding and analysis of COR-KT themes

Digital voice recordings of the interviews were transcribed verbatim and reviewed for accuracy. The transcripts were imported into QSR International's NVivo7 for coding and analysis. Thematic coding of all transcripts was completed by two independent coders. Themes were identified according to *a priori *categories derived from the KT and COR theory literature as well as newly emerging categories using NVivo7's node feature for each interview question. Disagreements between coders were resolved through discussion. NVivo7 reports generated the number of mentions of a given theme as well as a summary of quotes for each theme. SPSS 15.0 was used solely for the purpose of organization and to generate summaries of the data. Each of the themes generated through analysis using the NVivo7 software was assigned a numeric code. These data were entered into SPSS 15.0 along with the case study, type of respondent, and interview question number. The SPSS 15.0 output was used to generate frequency tables to assist in identifying predominant themes emerging from the interview data that have been summarized as text only in the results section of this paper.

## Results

### COR-KT themes

The findings of the multiple-case study are organized below according to the three COR-KT themes (Table 1). It should be noted that the interviews reached saturation wherein the general thematic content described by participants was consistent across the four cases.

#### COR-KT theme one: Resources are required for adaptation and change (in the context of research use)

For the most part, the organizational resources identified by interview participants were consistent with those identified in the literature scan (see the items with the subscript _ab _in Additional file 1, Table s1). Examples of these organizational resources include the accessibility of research evidence, the availability of incentives to use research evidence, opportunities for interactions between researchers and users of research, and the presence of a knowledge broker. Participants also identified organizational resources that had not been found in the literature (see the items with the subscript _c _in Additional file 1, Table s1); *e.g*., perceived economic efficiencies or limited costs (perceived or actual) associated with evidence-informed change, perceived need to act on research evidence, and satisfaction with prior research use efforts.

Organizational resources generally fit into four overlapping categories: organizational culture, human resources, economic resources, and condition resources (or states) within the organization (see Additional file 1, Table s1). To classify organizational resources and describe their conceptual relationship, we use the term vector to refer to the categories of organization resources [*e.g*., [416]]. We consider the four vectors separately, while acknowledging that quantitative methods and analyses are needed to determine the interrelatedness of the vectors. Within each vector are several dimensions (or groupings of similar elements). Components of the dimensions are described as elements. The vectors, dimensions, and elements within each dimension summarized below and are presented in Additional file 1, Table s1).

##### (1) Organizational culture

The organizational culture vector is defined by the norms and expectations concerning behavior and procedures related to research uptake within an organization [417]. Seven dimensions of organizational culture appear to be related to research uptake: 1.1. Policies and practices that guide research use; 1.2. Training to use research evidence; 1.3. Access to research evidence; 1.4. Organizational leadership; 1.5. Organizational flexibility; 1.6. Organizational buy-in; and 1.7. Organizational history.

##### (2) Human resources

The human resources vector is defined by characteristics of individuals within the organization. Characteristics of individuals greatly shape the organizational culture. Thus, it follows that specific characteristics of individuals may build resiliency and facilitate research uptake within health systems. Five dimensions of human resources appear to be related to research uptake: 2.1. Personal characteristics (*e.g*., attitudes, perceptions, motivation); 2.2. Skills/qualifications; 2.3 Activities; 2.4. The presence of change agents; and 2.5. Staffing.

##### (3) Economic resources

The economic resources vector is defined by the monetary or financial aspects of an organization. Four dimensions of economic resources are related to research uptake: 3.1 Budget constraints; 3.2. Spending flexibility; 3.3. Investment in research use activities; and 3.4. Economic dependency. Flexibility in how economic resources are allocated is particularly important. If economic resources are solely tied to fixed costs, with little opportunity to invest in evidence-informed change, organizations have limited capacity for research uptake.

##### (4) Condition resources

Current or situational time-limited conditions within the organization can affect its capacity for research use. Situational conditions can provide a catalyst for change and the opportunity to modify existing policies and/or policies. Alternatively, situational constraints may stifle research uptake. Three dimensions of condition resources related to research uptake are: 4.1 Time/Timing; 4.2. The absence of conflict; and 4.3. Opportunity.

### Resource needs as a function of the stages of research uptake

The results of the multiple case study support the notion that resource needs differ as a function of the stage of KT. Therefore, the discussion of resources and their importance is considered in the context of stages in the research uptake process. The type of participant (Researcher, Policy maker, or Practitioner) and case (UB, RSS, FCS, and DRS) are identified for each quote. A range of responses were selected purposefully to demonstrate similarities and differences that exist from different categories of participants across each of the four cases.

### Resource needs at the initial stages of research uptake

During the initial stages of research uptake, (*i.e*., at the discovery of and consideration of new research evidence) organizational culture were identified as important:

'I think organizational culture [is most critical in the beginning]. If ... particular organizations weren't open to partnering, even having the right people in the right places and the latitude to work on it within their positions, we wouldn't have moved [forward].' -- Researcher, FCS

'... I think the organizational culture recognized the value of research to practice. And they were given the opportunity to participate in decision making opportunities, like being part of working groups, the forum, being invited to the forum, and being as participants.' -- Policy maker, RSS

'The organization values, the leadership, the access, the exposure, are really pro-research, and we need to embark on this project because it is very important and our organization supports that.' -- Practitioner, RSS

Aspects of organizational culture that were perceived to initiate and support research use included the accessibility of research evidence, the presence of policies/infrastructure to support research use, and the belief in the benefits of research use:

'The organization invests in research-related articles, partly due to the affiliation with the [University].' -- Policy maker, RSS

'Opportunities do exist to foster learning and development of research skills.' -- Practioner, RSS

'I think there was more a feeling of freedom of moving between the political and the administrative sides of the organization.' -- Practitioner, RSS

'[Capacity building efforts were focused on] education and skills development versus addressing the root causes and looking at policy and system's change.' - Researcher, FCS

As evident from the above quotes, participants identified the need for investment in infrastructure and activities to support research use. Although the overall categories of resources identified were consistent across participants, some differences emerged in the types of resources that were identified as playing a prominent role in the initial stages of research uptake. Policy makers tended to emphasize the importance of flexibility within the organizational structure to make changes as new research evidence emerges. Competing demands and the need for equal distribution of resources were often reported to be a barrier to research uptake:

'In rural Nova Scotia, it is a struggle for resources. When you have limited resources, you have to be equitable about where to allocate funds. Do you put it here or there? Do you take it from here or there?' -- Policy maker, RSS

Practitioners tended to emphasize the need for sufficient time for advancing research use activities:

'Their [management] contributions and support would have been in the way of providing staff time to go to meeting and providing openings within their departmental meetings.' -- Practitioner, RSS

Researchers emphasized the importance of a new organizational receptivity to research use:

'There is an openness in the departments to hear about [research]. They are aware of it now. We went to a Policy Advisory Committee and presented it. And there is more and more with the [government] strategy.' -- Researcher, UB

### Resource needs at the implementation stage of research uptake

During the implementation stage of research uptake (*i.e*., once the decision has been made to act on research evidence), both human resources (*e.g*., champions, skilled staff who make a commitment sustain change) and economic resources (*e.g*., available resources, flexibility to reallocate economic resources) were reported as prominent themes in the uptake of research evidence. In particular, the presence of a champion or facilitator was considered to be among the most valuable resources in seeking the support of others for evidence-based change:

'One of the reasons that our work has been successful is that we've had some real champions leading the work.' -- Policy maker, FCS

'Under human resources, I think what was really key is now they have champions identified, with actually high respect in our organization. [Examples include a medical doctor and a stroke navigator].' -- Researcher, RSS

There were a few champions, I'll say, within the organization that were motivated and energized to help make some stroke care improvements.' -- Policy maker, RSS

'Having people in place to implement best practices: That was most important later on ... but to get there, you need the support of the organization.' -- Practitioner, RSS

Participants acknowledged that organizational culture is inextricably linked to characteristics of the individuals within the organization; most notably, the extent to which individuals are receptive to research/innovation, possess a research use orientation, and hold shared beliefs with others in the organization, and openness to collaboration (*e.g*., between researchers, decision makers, and practitioners):

'...Certainly in terms of readiness to proceed with trying out some of the best practices and the recommendations in the document, [our organization] was way far ahead of some of the [organizations in] other districts.' -- Policy maker, UB

'There are individuals in the organization who were really motivated and willing to adapt to change, and were really key players.' -- Researcher, UB

Aspects of economic resources that were reported to facilitate research uptake during the implementation stage included dedicated funds or the flexibility within the budget to reallocate funds. It was noted that change should occur with the realization of potential benefits and efficiencies from implementing new research evidence:

'Economic resources, I think there was definitely a realization that in order to improve stroke care to the recommended levels that were in the stroke strategy document, that money was going to be required. Not that is wasn't known all the way along, but I think they were thinking more in terms of what exactly do we need. Is it two OTs [occupational therapists] or three, or three speech pathologists, or what exactly is it? And starting to think about what dollars would have to go along with that.' -- Policy maker, RSS

Participants' comments illustrate the importance of time to establish and foster relationships between researchers, policy makers, and practitioners to effect change. Consequently, short-term collaborations may have limited impact if major systems change is required.

### Resource needs at the later stages of research uptake

During the later stages of sustaining newly implemented policies and/or practices, human resources and economic resources were considered to be essential for sustaining any changes to policy and/or practice resulting from research evidence:

'[We] need the resources to do it ...ultimately, dollars and human resources.' -- Researcher, RSS

Dedicated staff with a flexible workload to engage in change efforts were thought to play an important role in sustaining policy and/or practice changes in the later stages of research uptake. Economic resources including funds to sustain new policies and/or practices as well as a financially supportive system were considered to be increasingly important at this stage of research uptake, particularly when the changes were brought about through the course of a limited term funded research project.

#### COR-KT theme two: The threat of loss leads to the protection of assets

A central component of COR theory is the notion that the threat of resource loss results in the guarding of existing resources and risk aversion (*i.e*., pushback on research use). The fear of resource loss over potential benefits was documented in the four cases. All participants expressed some hesitation or resistance to engage in research use activities; however concerns differed among policy makers, practitioners, and researchers.

Policy makers were primarily concerned with the impact of dedicating resources to change policy and/or practice in one area to the detriment of other programs.:

'There was a fear that money would be taken away from other programs to be able to do this...' -- Policy maker, RSS

Practitioner concerns stemmed from having an unmanageable workload, decreased time, and role confusion:

'I am only one person! I was quite overwhelmed...where do you put your time and how do you make those decisions?' -- Practitioner, RSS

Concerns were expressed about the availability of health system support for the sustainability of a change that was being tested. However the concerns about loss varied as a function of stages in the KT pipeline. In the early stages:

'There were concerns about becoming involved because previous experience with research had left them unsatisfied [and led to a breakdown in trust]' -- Researcher, DRS

'Before you put the time and effort into it...is it sustainable? How are people going to respond to it? What directions will they be given? And will we be prepared for the potential outcomes in terms of resource allocation and capacity to respond.' - Policy maker, DRS

The later stages of a grant, termination of grant funding, and the coordination that comes with it, contributed to concerns about the sustainability of engaging in research use activities:

'All of a sudden, it was the end of the project, and the money was gone, the person was gone ... so a sense of disappointment that we didn't accomplish what we had hoped to ...' -- Community partner, DRS

'But what happened when the project ends is you no longer have that overarching coordination...[we] saw the differences ... it fell back to the provinces to implement and sustain the activity on a provincial basis because you lost that coordination.' -- Policy maker, DRS

'So if anything, after the money was done, all of these things became more strained.' -- Practitioner, DRS

In summary, worries over potential resource loss were heightened if participants had prior negative experiences with research. This issue was particularly salient if past research collaborations had resulted in losing a champion or losing skilled staff. Negative experiences with past research initiatives served to exacerbate resistance to research use and increased the scepticism concerning the benefits of changing practice and/or policy.

There were several marked differences between long-term and short-term projects involving research use. The salience of resource loss over the potential gains of research use was particularly strong among the participants in short-term projects. Participants conveyed a sense that there was insufficient time to develop a strong university-community partnership. Projects that received only short-term funding suffered from the lack of a strong research or policy champion. Participants reported that trust was not well-established between policy makers, community partners, and researchers. Limited communication between partners was perceived to decrease confidence in the recommended policy changes that resulted from the research. Interestingly, confidence in the research evidence was largely intertwined with the relationships between researchers, policy makers, practitioners, and community partners.

Involvement in long-term projects that connected directly to the development of health system changes seemed to build confidence among the service providers, allayed fears of resource loss, and increased capacity to act on research evidence. Participants in long-term projects reported that there was sufficient time to conduct the research, translate the findings, and facilitate system changes. Time, coupled with additional money and further involvement in partnerships appeared to generate greater receptivity to using evidence.

#### COR-KT theme three: Resources must be optimized for adaptation

All participants identified strategies that maximized the use of existing resources to gain buy-in. In particular, participants reported the value of a champion to create momentum among staff and buy in among decision makers:

'A champion makes all the difference in the world [in gaining buy-in and involvement].' -- Researcher, RSS

Ongoing education and training opportunities about the issue and approaches to addressing it, capitalizing on existing partnerships and collaborations served to bolster confidence in the ability to act on research evidence:

'[The principal investigator] had a history and a reputation for working in the area of food security ... provided credibility.'-- Researcher, FCS

'They encouraged...They allowed us, as clinicians, to go to the forum. And certainly several of us going involved with working groups.' -- Practitioner, RSS

'All new projects that are being built are being built to accommodate bicyclists as well. So if we are re-building a roadway, an existing roadway, if the opportunity exists, we widen the roadway to incorporate bike lanes ... bikeway projects would be tacked onto existing pre-planned, much larger roadway building projects.' -- Policy maker, UB

Together, these engagement strategies empowered individuals and teams within health systems and cultivated efficacy to enact evidence based change. Receptivity to research use was bolstered with confidence that improvements to service would result. Participants' comments reflect the importance of leveraging an existing resource -- even through a seemingly small act such as encouraging staff participation at a scheduled event -- and serves to create a culture shift and momentum towards implementing changes based on evidence. It appears that resource optimization occurs when threat of resource loss is countered with perceived benefits are associated with the outcomes of research use. In many cases, participants expressed excitement for resulting changes and reported an eagerness to engage in future research use activities:

'Benefits include the prevention of strokes among those who might otherwise have had strokes, potential for earlier and more effective treatment, and improved potential for quality of healthcare across the spectrum ... from prevention to rehabilitation.' -- Policy maker, RSS

'I think that we are going to gain a healthier population, a healthier future, a healthier environment. Not that we have gained it. These are long term things [that we will continue to act on].'-- Researcher, FCS

Although organizational resources can be optimized to enhance research uptake, there appears to be a threshold to optimization. Participants suggested that it is not as simple as 'making do with existing resources.' The provision of financial resources from the province that supported improvements to stroke care at the regional level helped to sustain momentum:

'Because of the money, we received equipment that enabled us to do a better job, increase our human resources, and become a more integrated team moving forward' -- Researcher, RSS

'So now that the province has awarded funding for the stroke program, I think there is excitement and commitment. And actually having resources really gives people an opportunity to do a lot of brainstorming and that kind of thing.' -- Policy maker, RSS

'If the Heart and Stroke Foundation hadn't pushed for the funding to go with it, the project might have been at the same place -- ending with no sustainability ... serendipitous.' -- Researcher, RSS

As evident from the multiple case study, there is some variation in how the COR-KT themes play out across the four cases. However, the four cases were consistent in providing evidence that the three COR-KT themes manifest in the health systems context and at varying stages of research uptake: Resources are required for research uptake; threat of resource loss leads to the protection of assets; and resources must be optimized for adaptation.

## Discussion

The purpose of this paper was to examine the potential applicability of COR theory to explaining health systems capacity for research use through the identification of resources needed for the uptake of research evidence into policy and/or practice and how resources, or a lack of them, influences receptivity to research use. A scan of the KT literature was conducted to identify the types of resources required for research uptake. A multiple case study was conducted to further classify the types of resources required for research uptake and validate the three central COR-KT themes in the context of research use in health systems.

Recent KT literature has focused on the application of cognitive-behavioral theories to individual practitioner behavior change (*e.g*., prescribing behavior) [418,419]. However, systems level changes require their own theoretical foundations. Consistent with the KT literature, our research provides evidence that organizational resources facilitate the uptake of research evidence (COR-KT theme one). We developed a taxonomy of organizational resources that favor research use within health systems, and thereby offer support for the initial COR theory theme as applied to KT (*i.e*., resources are required for research use).

Beyond identifying factors whose presence or absence affects research use, we provided preliminary support for the remaining COR-KT themes. The first COR-KT theme (*e.g*., resources are required for research use) was found to be widely documented in the KT literature. The value added by COR-KT theory to the extant KT literature stems from the remaining two COR-KT themes concerning the threat of resource loss in resource-challenged environments and how resources can be optimized to support research use; and the extension of COR theory to include change in resource needs as a function of stages in the research uptake process. The concept of loss is critically important in resource-challenged environments. Fear of resource loss can limit engagement in research use activities, and further contribute to the health systems gradient. Furthermore, our research suggests that overcoming resource constraints through the optimization of resources, even in resource-challenged environments, can support research use.

Our research extends both the literature on COR theory as well as the KT literature by revealing the potential importance of resource needs at various stages during the research uptake process. It may not be the total number of resources that builds capacity for research use. Rather, some resources may be more critical to research uptake than others, and some resources may be more or less critical at various stages of implementing the findings that derive from research. In light of this finding, what elements, if changed, will create better conditions for research uptake -- perhaps using a staged process? Although COR-KT theory considers the role of resource manipulations (and/or substitutions) in research uptake, their effects may be dependent upon the domain (or vector). For example can a resource in one vector (*e.g*., money) compensate for a lack of resources in another domain (*e.g*., leadership)?

## Limitations

This preliminary research provided some support for the value of COR-KT theory themes in the context of research use. However, the results of this research must be interpreted in light the following caveats. First, the multiple-case study was based on specific research projects. In these cases, the perceived benefits of being involved in a facilitated research project may have enhanced the group's motivation to engage in research use. Additionally, the promise of new resources (*e.g*., additional equipment, staff, prestige) may have provided much needed incentives to manipulate existing resources in the hopes of acquiring additional resources -- perhaps suggesting the need for external supports in place to engage potential research users. Furthermore, the research was conducted in a relatively economically depressed region of Canada. However, examination of how COR-KT principles play out in resource-challenged environments is needed on a more global level. Presumably, loss aversion would be exaggerated in regions with even fewer resources and require greater strategic investment to offset fear of resource loss.

A synthesis of the KT literature led to the development of a taxonomy of organizational resources central to research use within health systems and offers support for the notion that resources are critical for adaptation and change (*i.e*., COR theory principle one). For descriptive purposes, the organizational resources are presented as four distinct vectors. Further empirical testing of COR-KT theory in the health systems context is required. In the current research, we began to conceptualize the role of resources and tested some ideas using qualitative methods. Quantitative research is needed to validate the classification of resources presented in the taxonomy (see Additional file 1, Table s1). Specifically, factor analysis would determine the extent to which the four categories of organizational resources represent distinct constructs and confirm whether the indicators within each of the categories reliably measure the same construct. Additionally, a greater understanding of the wider determinants of the elements (*i.e*., those in the external environment) that influence the presence of resources within an organization is needed. The KT literature, and our research, acknowledges the importance of external resources (*e.g*., a political culture of receptivity to research and innovation, financial incentives and support for research and innovation, and favorable public attitudes toward research and innovation) in fostering the capacity for research uptake within an organization. Given that health systems are nested within larger systems, is it possible to classify external resources to the same extent as organizational resources?

## Summary

COR-KT theory may offer promise for understanding research uptake in resource-challenged contexts. However, it is not a prescription or formula for change. While the resulting COR-KT model may not capture the full complexity of the health systems environment, the theory development and research findings should stimulate a better understanding of the effect of resource limitations on research use and generate more thinking about practical strategies to optimize existing resources for evidence-informed health systems improvement. In particular, our research may contribute to the understanding of how changes to policy, programs, and/or practices can affect perceived threats to existing resources, with consideration of the disparities between high-, middle-, and low-resource countries and social groups [420,421].

## Competing interests

The authors declare that they have no financial or other competing interests.

## Authors' contributions

Authors are listed in order of contribution to this paper. CA was involved in this research as part of her postdoctoral fellowship at the Atlantic Health Promotion Research Centre, Dalhousie University. CA was responsible for the research design, the literature review, data collection and analysis, writing the manuscript, and contributed theoretical development. RFL and GW were Principal Investigators of this CIHR-funded program of research, conceived of original research idea, wrote the grant proposal, led the supervision of the research program, participated in the theoretical development and design of the program of research, and provided substantive written feedback on various drafts of this manuscript. SH, PM, RL, and ERB were co-investigators on the CIHR-funded program of research. SH provided considerable guidance with theoretical development and consultation concerning the adaptation of COR theory to KT. PM, RL, and ERB contributed to theory development and provided substantive feedback on various drafts of the manuscript. All authors read and approved the final manuscript.

## Authors' information

CA is an Assistant Professor (Research) at the Atlantic Health Promotion Research Centre, Faculty of Health Professions, Dalhousie University, 209 - 1535 Dresden Row, Halifax, NS B3J 3T1, Canada. RFL holds a Chair in Complex Chronic Disease and is the Scientific Director of Bridgepoint Collaboratory for Research and Innovation, Bridgepoint Health, University of Toronto, as well as a Canada Research Chair in Health Promotion, Professor, and Senior Scientist (on leave) at the Atlantic Health Promotion Research Centre, Dalhousie University, 209 - 1535 Dresden Row, Halifax, NS B3J 3T1, Canada. GW is an Assistant Professor in the School of Occupational Therapy, Dalhousie University, 5869 University Avenue, Halifax, NS B3H 3J5, Canada. SEH is a Professor and Chairperson of the Department of Behavioral Sciences at Rush University and Medical College, 1653 W. Congress Parkway, Chicago, ILL 60612-3244, USA. PJM is Director and Senior Researcher of the Manitoba Centre for Health Policy; Professor in the Department of Community Health Sciences, Faculty of Medicine, University of Manitoba; CIHR/PHAC Applied Public Health Chair (2008-2013); 408 - 727 McDermot Avenue, Winnipeg, MB R3E 3P5, Canada. RL is a Canada Research Chair in Globalization and Health Equity at the Institute of Population Health, and a Professor in the Department of Epidemiology and Community Medicine, University of Ottawa, 1 Stewart Street, Ottawa, ON K1N 6N5, Canada. ERB is the Director of the UCLA Center for Health Policy Research, and Professor in the School of Public Health, University of California, 10960 Wilshire Blvd., Suite 1550, Los Angeles, CA 90024, USA.

## Supplementary Material

Additional file 1 **Table s1: Taxonomy of organizational resources required for research use**. Click here for file
