# Supplementary material for: Conservation of resources theory and research use in health systems
Source: Implement Sci. 2010 Oct 20;5:79. doi: 10.1186/1748-5908-5-79 (PMC2978118; doi:10.1186/1748-5908-5-79)
Supplement: Additional file 1 — Table s1: Taxonomy of organizational resources required for research use. [file 1748-5908-5-79-S1.DOCX]

| **Table 2. Taxonomy of organizational resources required for research use** | | | |
| --- | --- | --- | --- |
|  | | | |
|  |  |  | Organizational Culture |
|  | 1.1 |  | Formal and/or Informal Policy and Practice |
|  |  | 1.1.1 | Recruitment and retention of innovators_a_ [108-110] |
|  |  | 1.1.2 | Positive recognition of innovators_a_[111-114] |
|  |  | 1.1.3 | Incentives to engage in research use activities_ab_ [115-121] |
|  |  | 1.1.4 | Regular evaluation of policies and/or practices based on research evidence_ab_ [121-132] |
|  |  | 1.1.5 | Research use is a key component of policy and practice_ab_ [133-141] |
|  |  | 1.1.6 | Investment in research use activities_ab_ [142-150] |
|  |  | 1.1.7 | Participation in research_ab_ [151-161] |
|  |  | 1.1.8 | Interactions between researchers and users_ab_ [162-188] |
|  |  | 1.1.9 | Involvement in decision making_ab_ [189-192] |
|  | 1.2 |  | Training to Use Research Evidence |
|  |  | 1.2.1 | Training opportunities in research use_a_ [193-196] |
|  | 1.3 |  | Access to Research Evidence |
|  |  | 1.3.1 | Access to research evidence_a_ [197-207] |
|  | 1.4 |  | Organizational Leadership |
|  |  | 1.4.1 | Leadership in research use_ab_ [208-218] |
|  |  | 1.4.2 | Management support for research informed change_ab_ [219-228] |
|  | 1.5 |  | Organizational Flexibility |
|  |  | 1.5.1 | Flexibility in the organizational structure to make changes and/or reallocate resources based on research evidence_c_ |
|  | 1.6 |  | Organizational Buy-In |
|  |  | 1.6.1 | Buy-in at the organizational level_ab_ [229-231] |
|  |  | 1.6.2 | Shared beliefs about the value of research_c_ |
|  | 1.7 |  | Organizational History |
|  |  | 1.7.1 | Organizational history of prior successes stemming from research use_ab_ [232-234] |
|  |  |  | Human Resources |
|  | 2.1 |  | Personal Characteristics |
|  |  | 2.1.1 | Motivation/vested interest in research use activities_ab_ [235] |
|  |  | 2.1.2 | Information seeking_a_ [236] |
|  |  | 2.1.3 | Trust in research(ers) _ab_ [237-238] |
|  |  | 2.1.4 | Receptivity to ideas and collaboration_ab_ [239-240] |
|  |  | 2.1.5 | Research evidence is not perceived to be a threat_ab_ [241-248] |
|  |  | 2.1.6 | Early adopters_a_ [249-252] |
|  |  | 2.1.7 | Perceived benefit of research use (for self or organization) _ab_ [253-255] |
|  |  | 2.1.8 | Perceived match between organizational priorities and research evidence_ab_ [256-260] |
|  |  | 2.1.9 | Perceived need to act on research evidence_c_ |
|  |  | 2.1.10 | Openness/receptivity to research, innovation, and change_ab_ [261-273] |
|  |  | 2.1.11 | Perceived reliability and validity of the research evidence_ab_ [274] |
|  |  | 2.1.12 | Willingness to cooperate with the implementation of new research evidence (even if it contradicts prior practice) _c_ |
|  |  | 2.1.13 | Satisfaction with prior research use_c_ |
|  |  | 2.1.14 | Perceived generalizability of the research evidence to the organization_c_ |
|  |  | 2.1.15 | Flexible administrators_a_ [275-277] |
|  | 2.2 |  | Skills/Qualifications |
|  |  | 2.2.1 | Skills to acquire, assess, and apply research to the local context_ab_ [278-296] |
|  |  | 2.2.2 | Knowledge and understanding of research_ab_ [297,298] |
|  |  | 2.2.3 | Ability to network/communicate with others_ab_ [299] |
|  |  | 2.2.4 | Training, education and research in applied social sciences or relevant field_a_ [300, 301] |
|  |  | 2.2.5 | Skills at evaluation_ab_ [302] |
|  |  | 2.2.6 | Team building skills_ab_ [303] |
|  |  | 2.2.7 | Awareness of research evidence_a_ [304-307] |
|  | 2.3 |  | Activities |
|  |  | 2.3.1 | Participation in decision making_ab_ [308-310] |
|  |  | 2.3.2 | Participation in research_a_ [311, 312] |
|  |  | 2.3.3 | Read publications/professional journals_a_ [313] |
|  | 2.4 |  | Presence of Change Agents |
|  |  | 2.4.1 | Person(s) to coordinate/ manage/ facilitate change_ab_ [314-316] |
|  |  | 2.4.2 | Presence of a knowledge broker_ab_ [317-329] |
|  |  | 2.4.3 | Presence of an advocate/ champion/ opinion leader to influence change_a_ [330-342] |
|  |  | 2.4.4 | Leadership_ab_ [343-345] |
|  |  | 2.4.5 | Expertise_a_ [346] |
|  |  | 2.4.6 | Absence of a gatekeeper(s) who prevent(s) research use_a_ [347-348] |
|  | 2.5 |  | Staffing |
|  |  | 2.5.1 | Low staff turnover_ab_ [349-350] |
|  |  | 2.5.2 | Manageable workload_ab_ [351-353] |
|  |  | 2.5.3 | Sufficient staff _ab_ [354-358] |
|  |  |  | Economic Resources |
|  | 3.1 |  | Budget Constraints |
|  |  | 3.1.1 | Sufficient money to cover the costs of acquiring evidence_a_ [359] |
|  |  | 3.1.2 | Sufficient money to implement and sustain evidence-based change_a_ [360-369] |
|  |  | 3.1.3 | Limited costs associated with evidence based change and/or realization of potential economic efficiencies from implementing new research evidence_c_ |
|  | 3.2 |  | Spending Flexibility |
|  |  | 3.2.1 | Slack resources/Flexibility to reallocate funds/discretionary spending/ Maintenance or redirection of financial reserves toward research use_ab_ [370-378] |
|  | 3.3 |  | Financial Investment in Research Use Activities |
|  |  | 3.3.1 | Economic incentives to produce research_a_ [379-386] |
|  |  | 3.3.2 | Financial investment in research related resources and activities_a_ [387] |
|  | 3.4 |  | Economic Dependency |
|  |  | 3.4.1 | Economic dependency (from outside sources) _a_ [388] |
|  |  |  |  |
|  |  |  | Condition Resources |
|  | 4.1 |  | Time/Timing |
|  |  | 4.1.1 | Timing of research relative to events in the user system_ab_ [389, 390] |
|  |  | 4.1.2 | Time to acquire, assess, assimilate, and implement new research evidence_ab_ [391-402] |
|  | 4.2 |  | Absence of Conflict/Competition |
|  |  | 4.2.1 | Limited conflict concerning research use_c_ |
|  |  | 4.2.2 | Limited competition for scarce resources within the organization_c_ |
|  |  | 4.2.3 | Decreased potential for further loss resulting from research use_c_ |
|  |  | 4.2.4 | Not operating in crisis management mode_c_ |
|  | 4.3 |  | Opportunity |
|  |  | 4.3.1 | Presence of a catalyst (crisis or innovation) that heightens the need for change triggers community dialogue_ab_ [403-408] |
|  |  | 4.3.2 | Organizational stability_ab_ [409-411] |
|  |  | 4.3.3 | Momentum_ab_ [412] |
| Note: Items with the subscript _a_ were identified by the literature scan. Items with the subscript _ab_ were identified by interview participants in the multiple case study and are consistent with those identified in the literature. Items with the subscript _c_ are new items identified by interview participants in the multiple case study. | | | |
